# Relationship between C:N/C:O Stoichiometry and Ecosystem Services in Managed Production Systems

**DOI:** 10.1371/journal.pone.0123869

**Published:** 2015-04-20

**Authors:** Bhim B. Ghaley, Harpinder S. Sandhu, John R. Porter

**Affiliations:** 1 Department of Plant and Environmental Sciences, Faculty of Science, University of Copenhagen, Højbakkegård Allé 30, 2630 Taastrup, Denmark; 2 Copenhagen Plant Science Centre, Faculty of Science, University of Copenhagen, Thorvaldsensvej 40, 1871 Frederiksberg C, Denmark; 3 School of the Environment, Flinders University, Adelaide SA 5001, Australia; 4 Natural Resources Institute, University of Greenwich, Chatam, United Kingdom; 5 Faculty of Agriculture and Life Sciences, Lincoln University, Lincoln 7647, New Zealand; University of California Davis, UNITED STATES

## Abstract

Land use and management intensity can influence provision of ecosystem services (ES). We argue that forest/agroforestry production systems are characterized by relatively higher C:O/C:N and ES value compared to arable production systems. Field investigations on C:N/C:O and 15 ES were determined in three diverse production systems: wheat monoculture (C_wheat_), a combined food and energy system (CFE) and a beech forest in Denmark. The C:N/C:O ratios were 194.1/1.68, 94.1/1.57 and 59.5/1.45 for beech forest, CFE and C_wheat_, respectively. The economic value of the non-marketed ES was also highest in beech forest (US$ 1089 ha^-1^ yr^-1^) followed by CFE (US$ 800 ha^-1^ yr^-1^) and C_wheat_ (US$ 339 ha^-1^ yr^-1^). The combined economic value was highest in the CFE (US$ 3143 ha^-1^ yr^-1^) as compared to the C_wheat_ (US$ 2767 ha^-1^ yr^-1^) and beech forest (US$ 2365 ha^-1^ yr^-1^). We argue that C:N/C:O can be used as a proxy of ES, particularly for the non-marketed ES, such as regulating, supporting and cultural services. These ES play a vital role in the sustainable production of food and energy. Therefore, they should be considered in decision making and developing appropriate policy responses for land use management.

## Introduction

The fluxes and stoichiometry of elements like carbon (C), oxygen (O) and nitrogen (N) in an ecosystem is dependent on the anthropogenic intervention (e.g., land use, management intensity etc.) coupled with other environmental factors like precipitation and climatic gradient [1, 2]. The insights into elemental stoichiometry can unravel ecological processes operating at different levels from field to landscape scale. These relationships also influence ecosystem structure, species composition and diversity, ecosystem functions and provision of ecosystem services (ES) [3–7]. ES are the benefits that humans derive from natural (forests) and managed (agriculture) ecosystems [8–10]. ES include processes such as, nutrient cycling, pollination, biological control of pests, water regulation, etc [11–14]. Different land use regimes can affect elemental stoichiometry and thereby soil biota population dynamics which in turn influences ES (for e.g., soil fertility) [1, 15]. ES are broadly classified as provisioning (food, fodder, energy production etc.), regulating (carbon sequestration, water holding capacity of soils etc.), supporting (nutrient cycling, primary productivity etc.) and cultural ES (aesthetic value, educational etc.) [9, 10]. With 60% of global ES on the decline, quantification, valuation and monitoring of ES have assumed immense importance due to their role in sustainable production of food, fodder and energy [16]. Global agriculture can play a significant role in improving ES through understanding the underpinning processes and the economic value of these services. Therefore, it is vital to understand the relationship between elemental ratios and ES in agroecosystems.

ES in high-input agricultural systems are affected due to altered C:N ratios via the external input of N fertilizers [2]. This influences the microbial population stoichiometry and affects crop yield and quality [17]. C:N is a fundamental indicator of biogeochemical cycles in ecosystems. Any shifts in C:N stoichiometry have wide ranging effects in terms of nutrient cycling, plant community composition and structure affecting ecosystem functions with impacts on biogeochemical cycles at local, national, regional and global scales [18]. This change in ecosystem functions can influence the economic value of ES at regional or global scale. In agro-ecosystems, various ES have been identified and quantified on the basis of outputs and biophysical assessments [11, 14, 19–23]. Some of these studies also estimated the combined economic value, comprising values of all four categories of ES (provisioning, regulating, supporting and cultural) [19–25]. However, there is lack of general understanding of the relationship between stoichiometric ratios and ES they influence. Moreover, economic valuation of ES especially from production systems is a challenging exercise with several shortcomings [26–27]. Many studies argue that the value of supporting services such as nutrient cycling and nitrogen fixation are inherently absorbed in the value of food produced on farmland therefore estimating individual supporting services may result in double counting [24, 25]. In this study, we try to avoid this by defining and classifying ES into intermediate and final services. We define that all ES are means towards ends and sometime they are end in themselves [27]. For example supporting and regulating services are intermediate services whereas final services comprise of provisioning and cultural ones [21–23, 27, 28]. In agroecosystems, intermediate services such as nitrogen fixation, nutrient cycling, water holding capacity etc result in final products (e.g., grains, fruits etc) and services. The value of intermediate services is not captured or paid by conventional market, these markets only pay for the food or output produced as market values are based on cost of inputs (land, labour, fuel, machinery use etc) and does not include other intermediate services that need to function before inputs are transformed into final products in the form of food or grain [21–23]. Unravelling of such relationships in agroecosystems can not only help develop more sustainable agriculture but also improve decision making for policy responses at the landscape level.

We investigated the C:N/C:O stoichiometry and their relationships to various categories of ES under three production systems. These were intensively managed conventional wheat (C_wheat_), 18-year old organically managed combined food and energy (CFE) system, and a 47-year old beech forest. These production systems thus provide a decreasing gradient of management intensity and level of external inputs. In this study, first, we defined three production systems and identified ES associated with these systems from the review of relevant literature. Second, we carried out field experiments to assess stoichiometric ratios and ES in three production systems. Third, we used these outputs to quantify and estimate the economic value of each ES in three systems. We then compared the economic values of ES with elemental ratios in three production systems. We conclude with discussion on options to sustainably manage production systems on the basis of their stoichiometric ratios and ES they influence.

## Materials and Methods

### Study sites

The trial sites for the study were located in two sites in eastern Denmark: one in Taastrup and another in Frederiksborg close to Hillerød. The trial site at Taastrup is an experimental farm (55°40'N, 12°18'E) under the Department of Plant and Environmental Sciences. At the Taastrup site, a combined food and energy production system (CFE), and a conventional wheat (*Triticum aestivum*) (C_wheat_) field were located. The Frederiksborg site (55°57'N, 12°21'E) is a European beech (*Fagus sylvatica* L.) forest, a long-term International Co-operative Program (ICP) Forest level II monitoring site. Both sites are owned and managed by the University of Copenhagen.

### C_wheat_

The C_wheat_ field is located adjacent to the CFE system. It was cropped with winter wheat and fertilizer and herbicide and pesticide inputs were applied according to standard practice in Denmark. The C_wheat_ site has been cultivated with annual cereal crops and grass for the last 15–20 years [29].

### CFE

**Fig 1:**
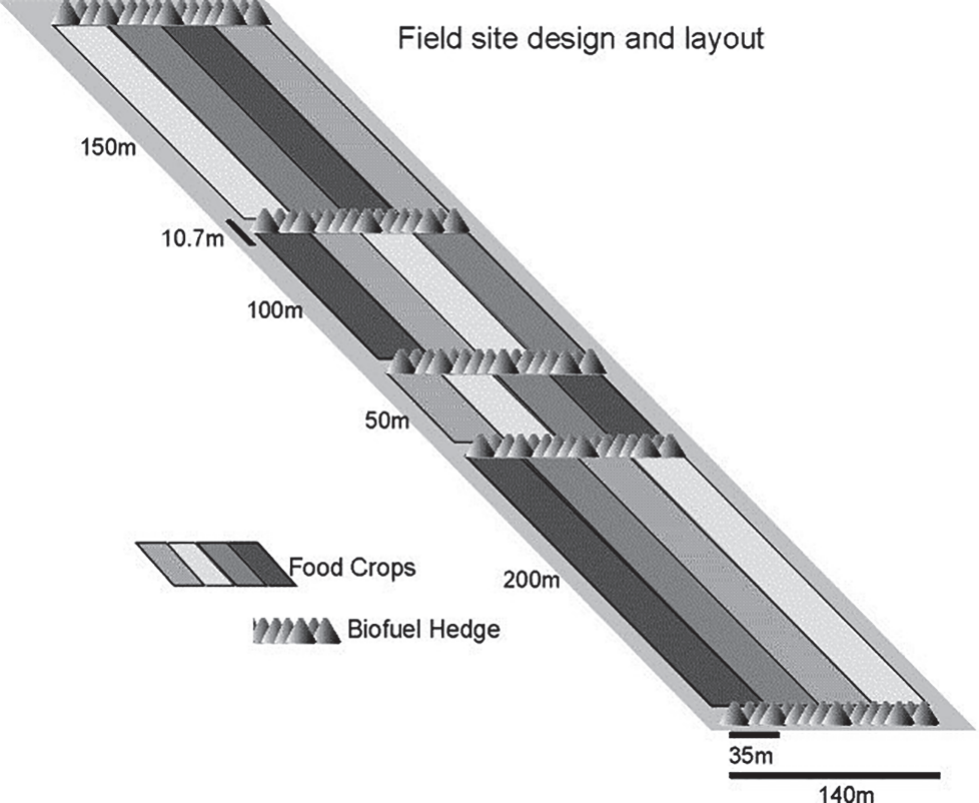
Schematic presentation of the combined food and energy system at the University of Copenhagen, Denmark.

CFE is laid out in 11.1 ha, consisting of food, fodder and bio-energy components and was established in 1995 and managed without industrially produced chemical inputs (Fig 1 [13]). In the CFE, the food components are winter wheat (CFE_wheat_), CFE_barley_ (*Hordeum vulgare*), CFE_oat_ (*Avena sativa*) and a fodder component consisting of CFE_ryegrass_ (*Lolium perenne*)/ lucerne (*Medicago sativa*) and CFE_willow_ consisting of ten rows of short rotation woody crops (SRWC) for bioenergy production. Of the ten SRWC rows, the six middle rows consist of three species (one double row each) of willow (*Salix viminalis* L.), *S*. *dasycladus* Wimmer and *S*. *triandra* x *cinerea* L.) bordered by one double row of common hazel (*Corylus avellana* L.) on one side and one double row of alder (*Alnus glutinosa* L.) Gaertner) on the other. Prior to 1995, the CFE site was continuously cropped with annual crops. The CFE willow belts cover about one hectare of the 11.1 ha of the whole CFE system.

### Beech forest

The beech site was formerly agricultural land and was re-afforested 47 years ago, and is characterised by loamy clay soil and high capacity to store plant available water [30].

Thus, there were seven production systems in the study, consisting of C_wheat_, beech forest and the five production systems embedded within the CFE system viz. CFE_wheat_, CFE_ryegrass/lucerne_, CFE_oat_, CFE_barley_ and CFE_willow_.

### Field sample collection, processing and analysis

Samples (e.g., soil, plants, roots etc.) from study sites were collected from March, 2011 to February, 2012. All samplings consisted of four replicates per production system. The samples collected were from above-ground (grain, straw, leaf, litter and wood), below-ground (root) and soil. Details of sample collection methods in the different production systems are provided in Table 1. Soil samples consisted of four bulk samples, each separated into five sub-samples, taken with a soil auger, to a depth of 25 cm. Sub-samples were air-dried at room temperature (25°C), sieved and any stones or macroscopic materials larger than 2mm were removed, followed by storage at -4°C until laboratory analysis. Plant samples were air-dried followed by oven drying at 80°C for 72 hours and dry weights were recorded. Samples were ground and the sub-samples taken.

**Table 1:** Overview of data collection and sampling frequency during the trial period.

Samples	Equipment required	Method	Trial plots
Grain sample	Scissors, plastic bags	sampling frame (3x2m^2^)	CFE wheat, CFE oat, CFE barley, C_wheat_
Straw sample	Scissors, plastic bags	sampling frame (3x2m^2^)	CFE wheat, CFE oat, CFE barley, C_wheat_, CFE ryegrass/lucerne
Root sample(0–25 cm)	Soil core sampler	<2mm diameter 10 root samples from 5 plants of one species	All production systems
Soil sample(0–25 cm)	Soil core sampler, cold box	sampling frame (0.5x0.5m^2^)	All production systems
Leaf sample	Scissors, plastic bags, cold box	20 leaves from 10 plants of one species (2 leaves per plant)	CFE willow and beech forest
Litter sample	Plastic bags	sampling frame (0.5x0.5m^2^)	CFE willow and beech forest
Wood core/branch sample	Wood core sampler	Samples from 5 individual plants of one species	CFE willow and beech forest

### Stoichiometric ratios

Soil and plant samples from each of the seven production systems were analysed for total carbon, oxygen and nitrogen with a CHNS/O analyzer, Thermo Fisher Scientific, Cambridge, UK [31]. C:N/C:O stoichiometry parameters for combined CFE system (CFE_average_) is weighted average across the five production systems embedded within the CFE based on 24% acreage each of CFE wheat, CFE barley, CFE oat, CFE ryegrass/Lucerne and 4% of CFE willow.

### ES measurements and economic valuation

Fifteen ES were identified based on previous literature in agroecosystems [13, 14, 21–23, 32]. These were classified into four categories: five provisioning services included grains (ES1), fodder (ES2), straw (ES3), wood (ES4) and bio-energy (ES5); five regulating services were water holding capacity (ES6), carbon sequestration (ES7), shelterbelt effects (ES8), soil erosion prevention (ES9), nitrogen fixation (ES10); four supporting services were pollination (ES11), biological pest control (ES12), nitrogen mineralization (ES13) and soil formation (ES14); one cultural ES identified was aesthetics (ES15). The ES measurements and economic valuation methods are detailed in an earlier study [13]. The economic value of each of the 15 ES was then estimated in USD (2012) by a combination of direct market valuation, avoided costs and value transfer methods [23]. These 15 ES were grouped into two: marketed ES (provisioning services) which are currently being traded in market and non-marketed ES (regulating, supporting and cultural services) which are currently not traded in the market. Provisioning and cultural ES are also the final products and services, whereas regulating and supporting services can be classified as intermediate services. The combined economic value from each production system is calculated by adding values of ES1 to ES15. Each of these 15 ES is either an intermediate or final services. Methods of each ES assessment are briefly discussed below,

**ES 1–5:** This ES is defined as the final product produced from each production system. For example, grains and straw from Cwheat system. CFE system produces wood chips and wood in addition to grains, straw and fodder. Beech forest provides wood only. In the CFE, grain and straw yields of CFE wheat, CFE barley and CFE oat were harvested in 2011 and their economic values were calculated based on the prevailing price of the grain (US$ 0.48 kg^-1^) and the straw (US$ 0.16 kg^-1^) in Denmark (www.farmtalonline.dk/accessed on 18.08.2013). Similarly, fodder yield of the ryegrass/lucerne was measured in 2011 and the prevailing price (US$ 0.16 kg^-1^) used for economic valuation. The CFE willow was harvested in 2011 and chipped and sold to a nearby heat generation plant and the economic value is the price received for the wood chippings (US$ 0.14 kg^-1^). In C_wheat_, the grain and straw yields were recorded and the corresponding grain (US$ 0.25 kg^-1^) and straw (US$ 0.12 kg^-1^) prices were used for economic valuation whereas in beech forest, the valuation was based on the annual wood volume production [33] and their corresponding share and prices of sawlogs (US$ 139 m^-3^), wood flooring(US$ 80 m^-3^), firewood (US$ 116 m^-3^) and wood chip production (US$ 139 m^-3^) [34].
**ES6:** Water holding capacity is defined as an ability of soil to hold moisture in between rainfall or irrigation. This is an important ES in production systems as it supports water availability to the plants/trees. The quantity of moisture available in the soil within plough layer of 25 cm for plant growth and crop production were measured with Time Domain Reflectometer (TDR). The economic valuation is based on the cost of extraction and application of the irrigation water (US$ 20 for 100 mm water) for cereal crop production (www.landrugs.info.dk/accessed on 18.08.2013).
**ES7:** Carbon sequestration is defined as the process to remove carbon from atmosphere in the form of above ground and below ground vegetation. In this study, we are using C market price to assess the economic value of C associated with different production systems. The aboveground and belowground biomass of the different annual/biannual crops (cereals, grass sward) and shelterbelt were determined ha^-1^ yr^-1^ based on the biomass sampling at harvest whereas in beech forest, yield tables were used for aboveground biomass estimation [35] ha^-1^ yr^-1^ by use of the biomass expansion factor reported for Danish beech stands [36]. Based on total biomass accumulation, 45% of the total biomass was considered to be carbon in cereals and grass sward whereas 50% was considered carbon in beech forest. The carbon was priced (US$ 10 ton^-1^) based on the prevailing price in the European Union Emissions Trading Scheme [37].
**ES8:** Soil erosion prevention as an ES is defined as process by which vegetation holds top soil against any potential erosion forces (by wind or water). The quantity of soil being prevented from erosion due to the extent of vegetation cover and type is based on a European-wide study [38] and the economic valuation is based on the price of soil (US$ 53.6 ton^-1^) available for vegetable gardening (www.lyngenatur-goedning.dk/accessed on 04.06.2013).
**ES9:** Shelterbelts are the barriers, usually made up of one or more rows of trees or shrubs, planted around the edges of fields to protect crops and animals against wind and also to provide shade. The shelterbelt effects are the increase in grain, straw and fodder yields due to the microclimate effects of enhanced moisture availability, reduced wind speed and crop damage [39, 40] and the prevailing grain and straw prices were used for economic valuation (www.farmtalonline.dk/accessed on 18.08.2013).
**ES10:** Nitrogen fixation is an important function of legumes that fixes atmospheric nitrogen into soil. Nitrogen fixed in the lucerne/ryegrass sward is based on a field investigation in Denmark [41] whereas the N fixation in alder is based on another study in Estonia [42]. The quantity of N fixed was multiplied by value of kg^-1^ nitrate fertilizer (US$ 0.48 kg^-1^) in Denmark, to arrive at the economic valuation.
**ES11:** Pollination is the process of transfer of pollen grains from anthers to stigmas and is generally carried insects (bees, wasps, beetles, flies, moths), vertebrates (birds, bats), wind and water. The dependence of important food crops on pollination makes this service crucial in agriculture. The economic value of pollination is based on number of beehives required for pollination and the cost of hiring the beehives (US$ 170 hive^-1^) based on an earlier investigation in CFE [23].
**ES12:** Biological control of insect pests is the process of control of pests by natural enemies such as predators and parasitoids. The economic value of biological control of pests is based on cost avoided for chemical control of pests in conventional wheat production system based on the field data collected in 2011.
**ES13:** Availability of plant nutrients by breakdown of organic material by soil micro and macro fauna is an important ES in production systems and is known as mineralization. The quantity of mineralized N was determined based on the extent of feeding activity of microbes on bait lamina probes. Bait lamina probes consisted of PVC strips (dimension 15–20 cm x 0.5 cm) with 16 holes (dia. 1mm), filled with bait material (mixture of cellulose powder, bran flakes, agar-agar etc) and exposed to biogenic decomposition process in the soil at 0–10 cm depth for measurement of the biological activity of the soil [43]. The economic valuation was based on the prevalent cost of nitrate fertilizer kg ^-1^ (US$ 0.48 kg^-1^) in Denmark.
**ES14:** Soil formation is an important ES for the maintenance of soil structure and fertility and is provided by earthworms. Soil formation is the quantity of topsoil formed based on the biomass of earthworms, sampled within 0.25 x 0.25 m^2^ plots. The mean biomass of an earthworm was 0.21 g (this study) and the earthworm biomass is considered equivalent to the quantity of top soil turned over ha^-1^ yr ^-1^ [21, 22]. The valuation is based on the price of the soil (US$ 53.6 ton^-1^) available for vegetable gardening in Denmark (www.lyngenatur-goedning.dk/accessed on 04.06.2013).
**ES15:** Cultural ES contribute to the maintenance of human health and well-being by providing recreation and aesthetics. For example enhanced landscapes by planting boundary vegetation contribute to improve aesthetics. Cultural ES is based on value transfer method and taken from regional studies [44, 45] where the socio-economic settings and aesthetic values are similar to Denmark. The range of values obtained from these studies was (US$ 176–332 ha^-1^yr^-1^).

### Statistical analysis

The elemental concentrations of C:N/C:O (mg/kg or %) in the plant and soil samples were converted into mmol/kg and molar (atomic) ratios were calculated. Before analysis, the molar ratios were log_10_ transformed to improve the variance homogeneity but ratios were back-transformed into actual values and units for reporting. The molar C:N/C:O ratios of each production system components (aboveground, belowground and soil) were calculated based on the concentration and quantity of the elements in different production system components in the above-ground (grain, straw, leaf, litter and wood), below-ground (root) and soil. The mean C:N/C:O molar ratios of production systems are weighted averages of the C:N/C:O contents in the above-ground, below-ground components and soil. The C:N/C:O stoichiometry differences were assessed under three management and production systems, viz. CFE_average_, C_wheat_ and beech. CFE_average_ production system has combination of woody (SRWC) and non-woody annual (cereal crops) and biannual (grass/sward) components. C_wheat_ is only non-woody annual crops whereas beech has only woody component. One-way ANOVA tests were run to assess the significance of differences in measured variables of C:N and C:O. Differences were considered significant if *P*≤0.05. Data were analysed with the Genstat software package (Genstat 8.1, 2005).

## Results

### C:N/C:O stoichiometry of above- and below-ground and soil components of the three production systems

Mean C:N molar ratios of the production system components in above-ground, below-ground components and soils (Table 2) were significantly different (*P*<0.05). Mean C:N ratios were highest in beech (194.1) due to relatively higher C:N ratios in the above and below-ground vegetation components. The mean C:N ratio of CFE (CFE_average_) (94.1) was significantly higher than C_wheat_ (59.5) but lower than the beech forest. Soil C:N ratios were significantly different *(P*<0.05) and increased in the order; C_wheat_ (12.2) < CFE_average_ (13.1) < beech (15.0) (Table 2). The root C:N ratio was highest in the beech (150.5) and the lowest in C_wheat_.

**Table 2:** C:N stoichiometry of aboveground (grain, wood, straw/fodder, leaf, litter), below-ground (root) and soil in combined food and energy (CFE) system, conventional wheat (C_wheat_) and beech forest.

Production system		C:N molar ratios			C:O molar ratios			
	Soil	Root	Above-ground	Mean	Soil	Root	Above-ground	Mean
CFE_average_	13.1	84.2	96.8	94.1	1.08	2.00	1.66	1.57
C_wheat_	12.2	73.0	76.3	59.5	0.94	2.17	1.34	1.45
Beech	15.0	150.5	278.9	194.1	0.92	2.05	1.74	1.68
*P*_(0.05)_	0.7	34.9	63.6	34.0	0.07	0.16	0.25	0.08

Comparing CFE_average_, C_wheat_ and beech, mean C:O ratios were significantly higher *(P*<0.05) in beech and CFE_average_ compared to C_wheat_ (1.45) (Table 2). The soil C:O ratios were lowest in beech (0.92) and CFE_avergae_ had significantly higher soil C:O ratios compared to C_wheat_ and beech forest soil. The root C:O ratio was highest in C_wheat_ (2.17) whereas above-ground C:O ratios were highest in beech and lowest in C_wheat_.

### ES quantification and valuation

The biophysical quantification and the economic value of 15 ES in the three production systems are provided in Table 3. Compared to beech and C_wheat_, CFE is characterised by production of multiple provisioning ES in terms of grain, straw, fodder and wood chip production and higher price premiums were received for CFE products due to organic nature of production. More than two-fold higher grain and straw yields in C_wheat_, compared to CFE, resulted in highest economic value for the provisioning ES among the production systems.

**Table 3:** Biophysical quantification and economic valuation of ES in CFE _average_, C_wheat_ and beech forest at the experimental sites in Denmark.

Ecosystem services	Units	CFE	C_wheat_	Beech forest	CFE	C_wheat_	Beech forest
		Biophysical quantification			Economic value (US$ ha^-1^yr^-1^)		
*Provisioning*							
Grains	kg ha^-1^yr^-1^	3228	7341	0	1553	1835	0
Straw	kg ha^-1^yr^-1^	2859	4941	0	457	593	0
Fodder	kg ha^-1^yr^-1^	1898	0	0	303	0	0
Wood chips	kg ha^-1^yr^-1^	217	0	0	30	0	0
Wood	kg ha^-1^yr^-1^	0	0	6900	0	0	1276
					2343	2428	1276
*Regulating*							
Water holding capacity	Mm	411	283	193	82	57	39
Carbon sequestration	ton ha^-1^yr^-1^	5	10	4	51	98	40
Erosion prevention	ton ha^-1^yr^-1^	1	0	3.3	53	0	177
Shelterbelt effects							
Grain increase	kg ha^-1^yr^-1^	446	0	473	214	0	228
Straw increase	kg ha^-1^yr^-1^	732	0	1050	121	0	173
Nitrogen fixation	kg ha^-1^yr^-1^	20	0	0	9	0	0
					530	155	657
*Supporting*							
Mineralized N	kg ha^-1^yr^-1^	108	64	192	52	31	92
Pollination		0	0	0	24	0	0
Pest control		0	0	0	4	0	0
Soil formation							
Soil formed	ton ha^-1^yr^-1^	0.3	0.3	0.2	14	15	8
					94	46	100
*Cultural*							
Aesthetics					176	138	332
Combined value	US$ha^-1^yr^-1^				3143	2767	2365
Non-marketed	US$ha^-1^yr^-1^				800	339	1089

Combined value of ES was highest in CFE_average_ valued at US$ 3143 ha^-1^ year^-1^ followed by C_wheat_ and beech forest at US$ 2767 and 2365 ha^-1^ year^-1^, respectively (Table 3). However, the non-marketed component was highest in beech forest followed by CFE_avergae_ and C_wheat_.

### ES and C:N/C:O ratios

**Fig 2:**
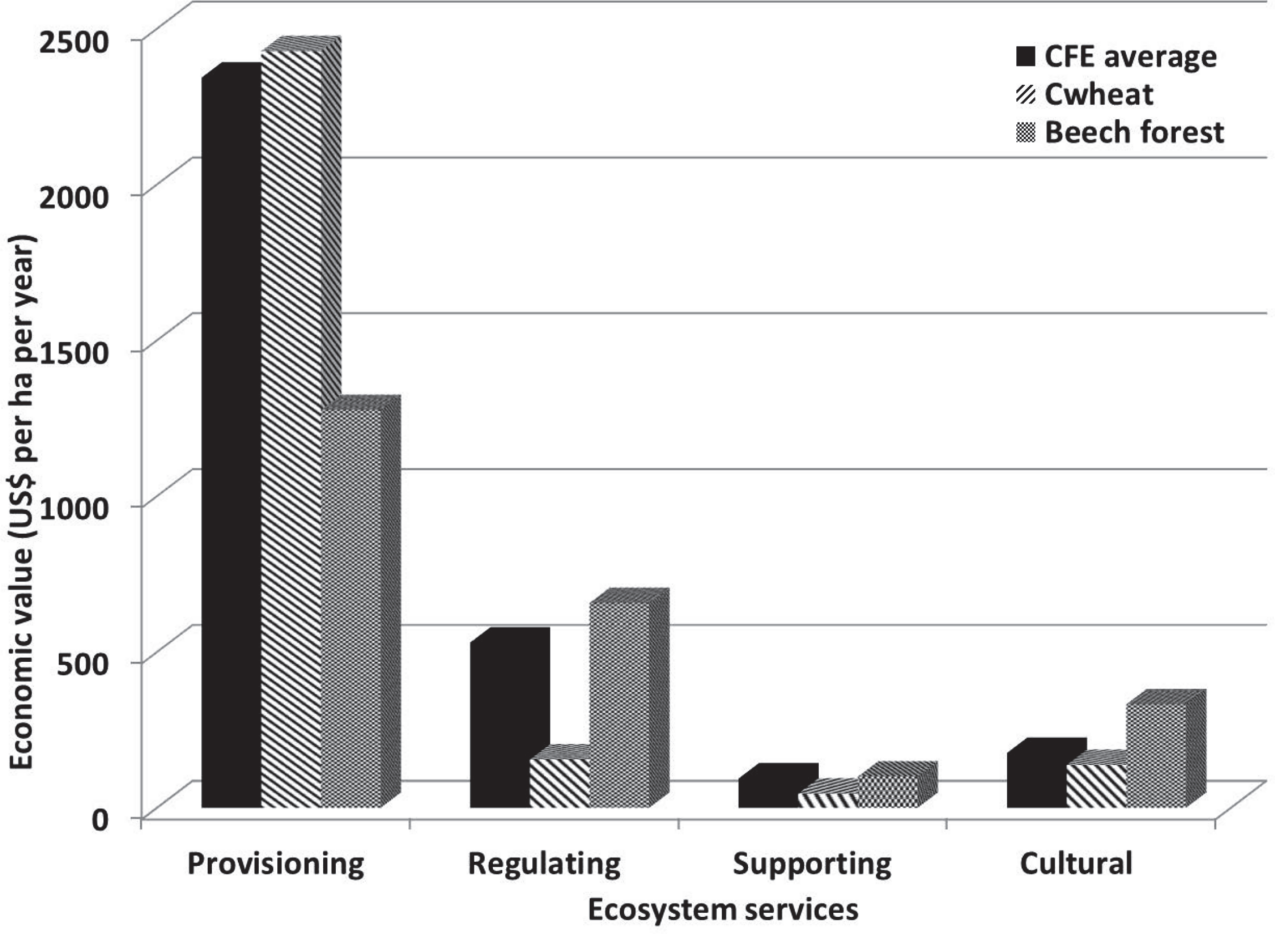
Economic values of four categories of ecosystem services in three production systems; C_wheat_, CFE_avearge_ and beech forest.

**Fig 3:**
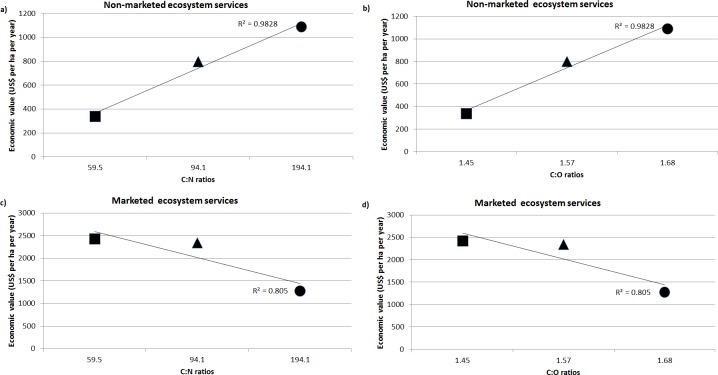
Relationships (trend line) between the economic value of non-marketed and marketed ecosystem services with C:N/C:O ratios in three production systems (■: C_wheat_, ▲: CFE_average_, ●: beech forest).

Out of the 15 ES assessed in the study, the economic value of provisioning services was highest in C_wheat_ followed by CFE_average_ and beech forest (Fig 2). Beech forest has the higher ratios of C:N/C:O followed by CFE_average_ and C_wheat_. An interesting trend was found out that systems with higher C:N/C:O ratios provided higher number of regulating, supporting and cultural ES. As there were no provisioning services identified in beech forest that can be consumed as food (grains or fodder), therefore, we compared only CFE_average_ and C_wheat_. Whereas, higher C:N/C:O ratios increased provisioning ES in CFE_average_ than the C_wheat_. The relationships between economic values and C:N/C:O ratios are further summarised in Fig 3. Economic value of non-marketed ES increases with increasing C:N/C:O ratios (Fig 3A and 3B: R^2^ = 0.98) whereas marketed ES decreases significantly (Fig 3C and 3D: R^2^ = 0.80).

## Discussion

A higher soil C:N ratio in beech compared to the other production systems in the study is congruent with data showing C:N in forest soils to be higher (14.5) compared to grassland (13.8) [46]. The lower soil C:N ratios in the C_wheat_ compared to the beech forest in our study corresponds to a pattern of lower C:N ratios in intensively managed agricultural system (10.8) compared to woodland (11.8) in a previous study [2]. The soil C:N stoichiometric ratios found in our study compared well to C:N ratios found in global soil data (186:13), soils in China (134:9) [47], forest [48] and in grassland [49].

The C:N/C:O elemental signature of a production system is the combined result of a number of processes with different rates of respiration and metabolism: soil parent material weathering, plant material return (litter fall, crop residues), plant uptake, transformations in the soil, surface/lateral and deep transport, soil erosion, atmospheric deposition and anthropogenic activities [47–49]. We hypothesized that the production systems at different gradients of human-induced disturbance (intensity in land use) differ in C:N/C:O stoichiometry and have influence on the biophysical measures and hence the value of ES. Our study demonstrated that non-marketed ES is higher in production systems like beech with minimal human disturbance and decreases along a gradient of human interference from extensive management to intensively managed production systems (C_wheat_) [21, 22]. In contrast, provisioning ES was found to be highest in C_wheat_ and lowest in beech demonstrating the effects of production system and management regimes ranging from ecologically-friendly practice (CFE) to conventional wheat monoculture augmented with substantial external inputs [23]. Non-marketed ES are indispensable inputs for sustainable intensification of agroecosystems, the substitution of which will have substantial social costs on the society [50, 51]. For example, in organically managed CFE, we reap the benefits of enhanced soil water holding capacity, reduced soil erosion, increased grain and straw yield due to shelter belt effects whereas in intensively managed C_wheat_, the above ES are either non-existent or minimum. Moreover, lack of provision of these ES would entail costs to the farmer and to the society as a whole [10]. Given the significance of non-marketed ES for sustainable intensification, loss of these services will increase our dependence on external inputs, which are energy-intensive and place additional demand on the environmental resources [10].

The notion of ES is to capture the value of all individual services (intermediate and final) so that sustainable systems can be developed unlike the current ones where we add fossil fuel based external inputs that result in high cost to human health and damage to the environment [50]. Economic valuation of ES in this study captures these intermediate and final services and classifies them into marketed and non-marketed ES based on previous studies [21–23]. Usually it is believed that the farm products includes the value of ES, but it is not the case [23]. For economic valuation, products and services can be counted individually and then added together to get an estimate of combined goods and services [21–23]. Here, we counted each ES at the point where it provides benefits, and we tried to avoid double counting by not counting twice the inputs to a product or service. For example, the carbon from crop residues that is incorporated into the soil and counted as a carbon sequestration service (ES7) is not included in determining the market value of the provisioning services (ES1-5). Double counting is a key area of debate in ES valuation. This study avoids double counting and reflects the combined economic value of ES in three production systems. These economic values are based on the functions they perform which is dependent on C:N/C:O ratios. As the C:N/C:O increased from C_wheat_ to beech, non-marketed ES increased whereas marketed ES decreased exhibiting that ES portfolio of marketed and non-marketed shifts with production systems and management regimes (Fig 3). The relationship demonstrated an empirical link between ES portfolio and C stoichiometry. However, the economic value of ES is also dependent on market volatility. Although our study ignored the long term impacts of market price fluctuations, the snap shot of the economic values are reliable estimates to support our conclusions. With global decline in ES, we need robust indicators at the field scale to assess the share of marketed and non-marketed ES under diverse management regimes so that informed decisions can be incorporated to maintain ES-rich production systems [23]. The findings demonstrated that carbon-dense production systems (CFE, beech forest) are more conducive for non-marketed ES provision than the low carbon dense systems. Since carbon sequestration in vegetation and soils are considered as one of the most cost-effective methods to sequester carbon [52, 53], production systems like beech and CFE with high C:N/C:O ratios have dual advantages for carbon sequestration and ES provision for the sustainable co-production of food, fodder and energy, without compromising on the ecological integrity of the environment. With ES-based approach to land management gaining priority at different scales of operation (local, national, EU, regional and global etc.), proxy like C:N/C:O can be used as an indicator for ES provision. In order to establish the robustness of the C:N/C:O as ES proxy, more studies need to be carried out in different socio-economic contexts and under various management systems. This could provide insights into new research direction for ES provision and sustainable management of natural resources.

## Conclusions

This study confirms that production systems with least management intensity and minimum inputs demonstrate higher stoichiometric ratios (Table 2). Increasing intensity of management and external inputs results in lower ratios. These trends are also consistent when the value of non-marketed components of ES is compared in three production systems (Fig 3A and 3B). These non-marketed ES are vital for the generation of provisioning services [10–14]. Valuation of all intermediate and final products and services can help in developing sustainable production systems. The study demonstrates that C:N/C:O ratios can be used cautiously as an indicator of ES under different management systems. However, further investigations are required to confirm these relationships. For example comparing arable agriculture under different management, such as conventional, organic systems or mixed cropping and livestock operations. These further studies can inform development of sustainable production of food, feed and energy for the growing demand and land use management.
